# Crystal structure of (5-{3-[(1,4,7,10,13-penta­oxa-16-aza­cyclo­octa­decan-16-yl)carbonyl­amino]­phen­yl}-10,15,20-tri­phenyl­porphyrinato)cobalt(II)

**DOI:** 10.1107/S2056989017007745

**Published:** 2017-06-07

**Authors:** Qi Wu, Mingrui He, Jianfeng Li

**Affiliations:** a College of Material Science and Opto-electronic Technology, University of Chinese Academy of Sciences, Yanqi Lake, Huairou District, Beijing 101408, People’s Republic of China

**Keywords:** crystal structure, crown ether-porphyrin, cobalt(II), hydrogen bonding

## Abstract

In the title compound, [Co(C_57_H_52_N_6_O_6_)], the central Co^II^ atom is coordinated by four pyrrole N atoms of the porphyrin core and one O atom of the crown ether. The complex has a distorted porphyrin core, with mean absolute core-atom displacements of 0.14 (10) (*C*_a_), 0.20 (10) (*C*_b_), 0.24 (4) (*C*_m_) and 0.18 (10) Å (*C*_av_), respectively. The axial Co—O bond length is 2.3380 (15) and the average Co—N_p_ bond length is 1.968 (5) Å. Intra­molecular N—H⋯O and inter­molecular C—H⋯π inter­actions are observed.

## Abstract 2

In the title compound, the central Co^II^ atom is coordinated by four pyrrole N atoms of the porphyrin core and one O atom of the crown ether. Intra­molecular N—H⋯O and inter­molecular C—H⋯π inter­actions are observed

## Chemical context   

Crown ether-porphyrinates have been developed to mimic the active site of the cytochrome *c* oxidase. There have been some reports on the single-crystal structures of crown ether-porphyrinates, including chlorido­[5^2^-*N*-(4-aza-18-crown-6)methyl-5^4^,10^4^,15^4^,20^4^-tetra-*tert*-butyl-5^6^-methyl-5,10,15,20-tetra­phenyl­porphyrinato]iron(III) (Dürr *et al.*, 2007 ▸), 5,15-{2,2′-[3,3′-(1,4,10,13-tetra­oxa-7,16- di­aza­cyclo­octa­decan-7,16-di­yl)dipropionamido]­phen­yl}-2,8,12,18-tetra­ethyl-3,7,13,17-tetra­methyl­porphyrin and the corresponding zinc(II) compounds (Comte *et al.*, 1998 ▸), 1,4,10,13-tetra­oxa-7,16-di­aza-cyclo­octa­decane-7,16-di­carb­oxy­lic acid{2,20-[10,20-bis-(3,5-di­meth­oxy­phen­yl)porphyrin-α-5,15-di­yl]diphen­yl}di­amide and the corresponding zinc(II) and lead(II) compounds (Halime *et al.*, 2007 ▸), aqua­{5,15,10,20-bis­[bis­(2-(1,10-di­aza-18-crown-6-1,10-di­yl)carbonyl­amino­phen­yl]porphyrinato}zinc(II) (Michaudet *et al.*, 2000 ▸). Herein, the crystal structure of a cobalt(II) porphyrin complex, (5-{3-[(1,4,7,10,13-penta­oxa-16-aza­cyclo­octa­decan-16-yl)carbonyl­amino]­phen­yl}-10,15,20-tri­phenyl­porphyrinato)cobalt(II), is reported.
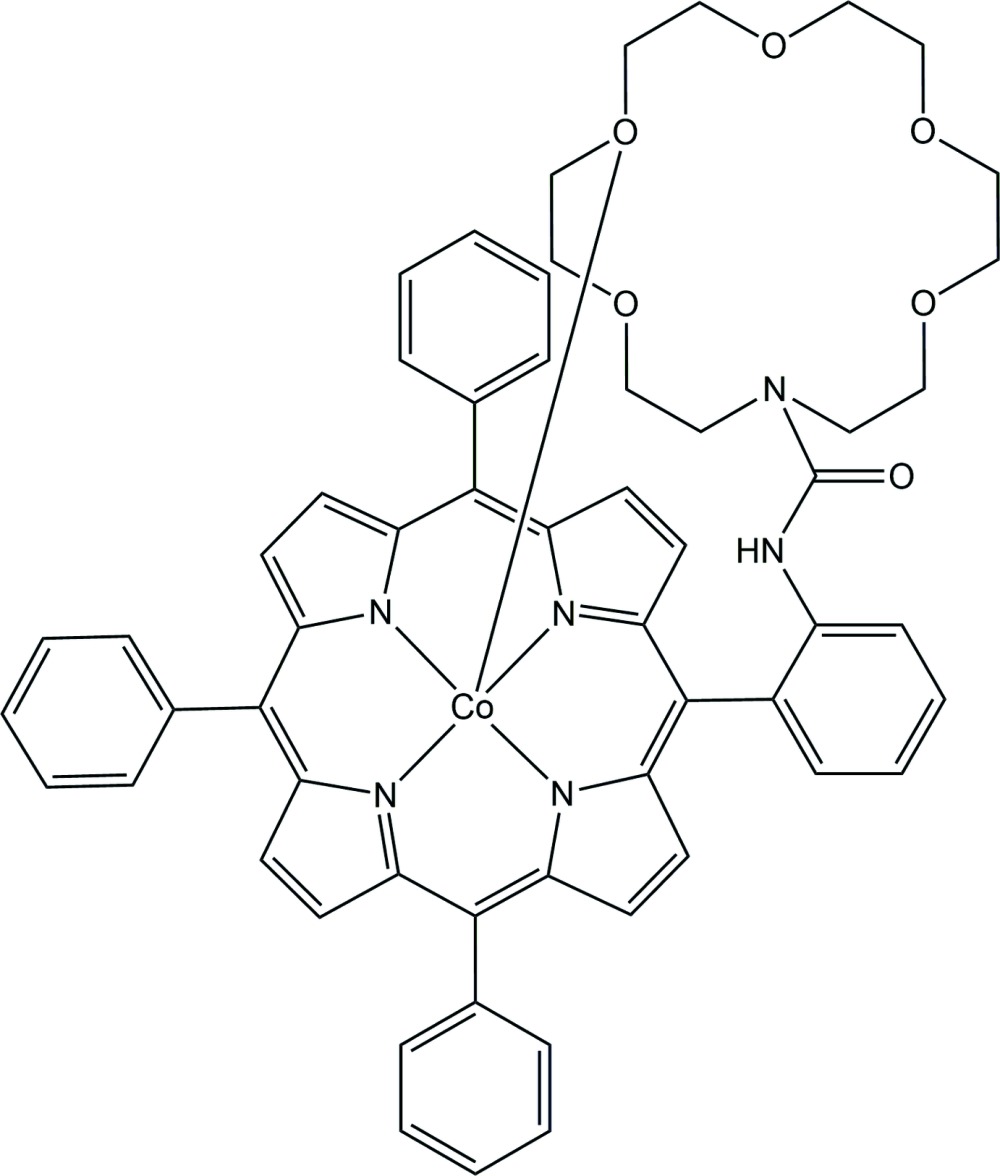


## Structural commentary   

**Figure 1:**
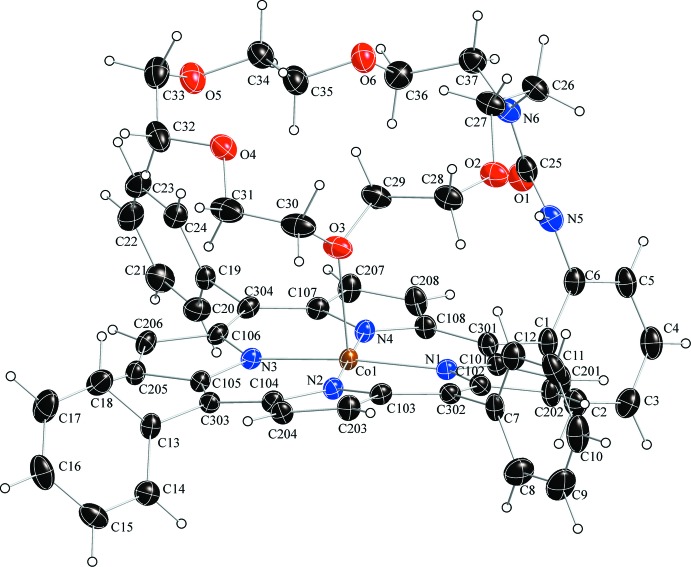
The mol­ecular structure of the title compound, with displacement ellipsoids drawn at the 50% probability level.

**Figure 2:**
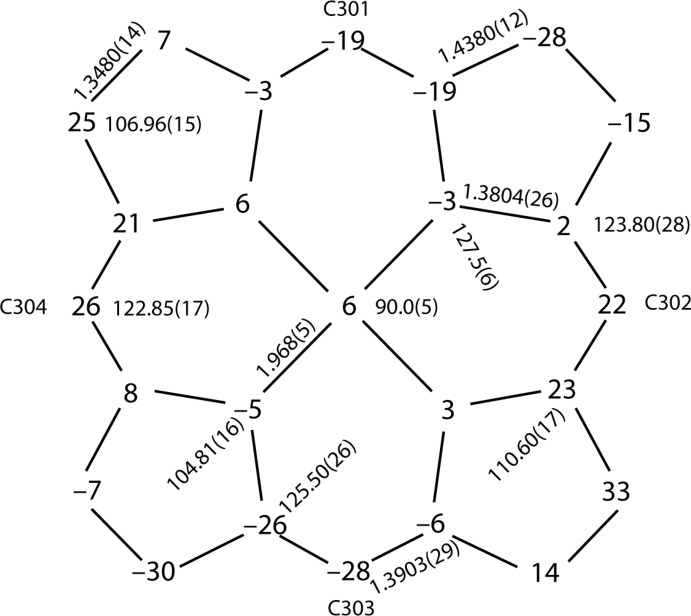
Diagrams of the porphyrin core of the title compound. Averaged values of the chemically unique bond lengths (in Å) and angles (in °) are shown. The numbers in parentheses are the s.u. values calculated on the assumption that the averaged values are all drawn from the same population. The perpendicular displacements (in units of 0.01 Å) of the porphyrin core atoms from the 24-atom mean plane are also displayed. Positive values of the displacements are towards the oxygen atom as the axial ligand.

In the crystal of the title compound (Fig. 1 ▸), the asymmetric unit contains one five-coordinate single-crowned porphyrin in which the oxygen atom (O3) of the crown ether ligates to the central cobalt(II) atom. Additional qu­anti­tative information on the structure is given in Fig. 2 ▸, which displays the detailed displacement of each porphyrin core atom (in units of 0.01 Å) from the 24-atom mean plane. Averaged values of the chemically unique bond lengths (in Å) and angles (in °) are also shown. The average Co—N_p_ (N_p_ is the porphyrin nitro­gen atom) bond length is 1.968 (5), in the narrow range of 1.958 (2)–1.969 (2) Å reported by Dey & Rath (2014 ▸). The axial Co—O (O is the crown ether oxygen atom) bond length is 2.3380 (15) Å, slightly longer than the values of 2.230 (5) and 2.2724 (7) Å found in the structures of [Co^II^(TDPMP)(CH_3_OH)] [TDPMP = 5,10,15,20-tetra­kis­(di­phenyl­meth­yl)porphyrin; Runge *et al.*, 1999 ▸] and [Co^II^(amtpp)]_2_ (amtpp = 5^2^-amidato-5,10,15,20-tetra­phenyl­porphyrin; Yamanishi *et al.*, 2011 ▸), respectively.

The cobalt(II) cation is displaced slightly from the porphyrin core to the axial ligand, as illustrated by the displacement of the metal atom from the 24-atom mean plane (Δ_24_ = 0.06 Å). The title compound shows a distorted porphyrin core conformation. The mean absolute core-atom displacements *C*_a_, *C*_b_, *C*_m_ and *C*_av_ are 0.14 (10), 0.20 (10), 0.24 (4) and 0.18 (10) Å, respectively.

An intra­molecular N—H⋯O inter­action is found between one of the oxygen atoms (O2) of the crown ether and the nitro­gen atom (N5) of the amide linker. The distance between O2 and N5 is 2.886 (2) (Table 1 ▸), consistent with the range (2.70–3.05 Å) suggested for the existence of N—H⋯O hydrogen bonding (Bertolasi *et al.*, 1995 ▸).

## Supra­molecular features   

**Figure 3:**
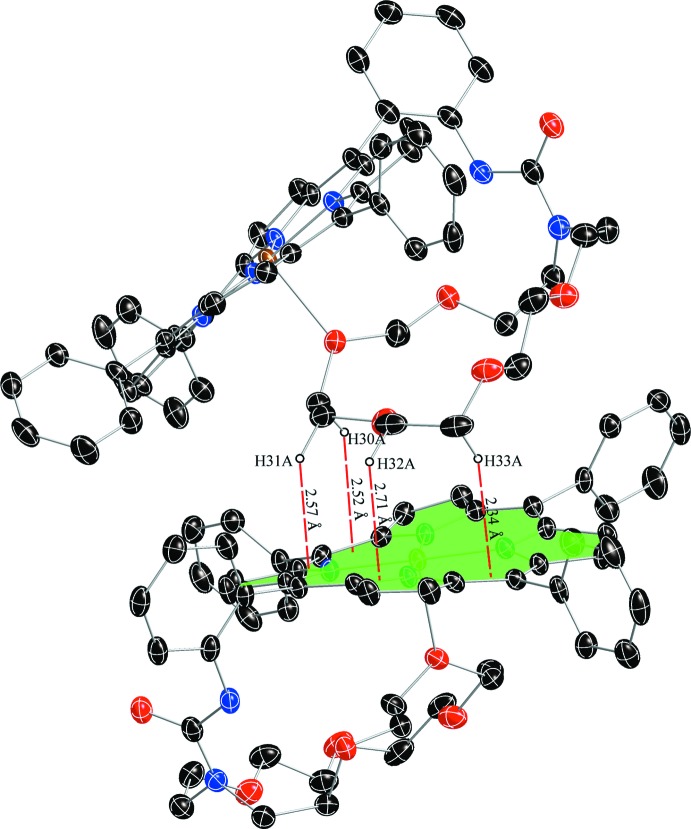
The C—H⋯π inter­actions in the title compound. Dashed lines show the distances between hydrogen atoms of the crown ether and the porphyrin core plane. Other atoms have been omitted for clarity.

**Figure 4:**
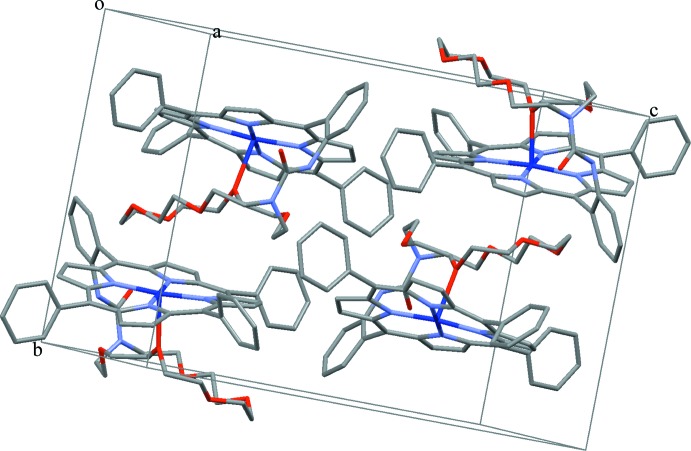
A view of the mol­ecular packing of the title compound in the crystal structure. H atoms have been omitted for clarity.

In the title compound, as seen in Fig. 3 ▸, the distances between the hydrogen atoms (H30*A*, H31*A*, H32*A*, H33*A*) of the crown ether and the plane of the neighbouring porphyrin core are 2.52, 2.57, 2.71 and 2.34 Å, all of which are smaller than 2.9 Å, a limit suggested for the existence of C—H⋯π inter­actions (Takahashi *et al.*, 2001 ▸). The molecular packing is shown in Fig. 4 ▸.

## Synthesis and crystallization   

General procedure: All reactions were carried out using standard Schlenk techniques under argon unless otherwise noted. Tetra­hydro­furan (THF) was distilled over sodium/benzo­phenone, hexa­nes over potassium-sodium alloy and di­chloro­methane (CH_2_Cl_2_) over calcium hydride. 5^2^-Amino­phenyl-5,10,15,20-tetra­phenyl­porphyrin was prepared according to the reported method (Lembo *et al.*, 2009 ▸).

### Synthesis of 5-{3-[(1,4,7,10,13-penta­oxa-16-aza­cyclo­octa­decan-16-yl)carbonyl­amino]­phen­yl}-10,15,20-tri­phenyl­porphyrin   

5-{3-[(1,4,7,10,13-penta­oxa-16-aza­cyclo­octa­decan-16-yl)carb­onyl­amino]­phen­yl}-10,15,20-tri­phenyl­porphyrin was prepared according to a modification of the reported methods (Wu & Starnes, 2012 ▸; Collman *et al.*, 1998 ▸).

Triphosgene (220 mg, 0.74 mmol) was added to a THF (150 mL) solution of 5^2^-amino­phenyl-5,10,15,20-tetra­phenyl­porphyrin (1.472 g, 2.3 mmol) and tri­ethyl­amine (Et_3_N, 0.7 mL) at 273 K. The mixture was stirred for 1 h and evaporated to dryness under vacuum. A CH_2_Cl_2_ (150 mL) solution of 1-aza-18-crown-6 (0.66 g, 2.5 mmol) and Et_3_N (0.3 mL) was added to the resulting solid stepwise. After overnight stirring, the solution was evaporated. The porphyrin product (1.48 g, 70%) was obtained by chromatography on a silica gel column (CH_2_Cl_2_).

### Synthesis of (5-{3-[(1,4,7,10,13-penta­oxa-16-aza­cyclo­octa­decan-16-yl)carbonyl­amino]­phen­yl}-10,15,20-tri­phenyl­porphyrinato)cobalt(II)   

(5-{3-[(1,4,7,10,13-Penta­oxa-16-aza­cyclo­octa­decan-16-yl)carbonyl­amino]­phen­yl}-10,15,20-tri­phenyl­porphyrinato)cobalt(II) was prepared according to a modification of the reported method (Adler *et al.*, 1970 ▸).

Dried CoCl_2_ (1.68 g, 12.9 mmol) was added to a THF (150 mL) solution of 5^2^-*N*-(4-aza-18-crown-6)acyl­amino-5,10,15,20-tetra­phenyl­porphyrin (0.6 g, 0.65 mmol). The mixture was refluxed for 3 h until the reaction was complete (monitored by TLC). The solution was extracted with CH_2_Cl_2_, washed with distilled water 2–3 times. After drying over Na_2_SO_4_ and filtration, the solvent was removed by rotoevaporation. The cobalt porphyrin product (0.52 g, 92%) was obtained by chromatography on a silica gel column (chloro­form: methanol; 20:1). The title crystal was obtained in a THF solution with hexa­nes as non-solvent.

## Refinement   

Crystal data, data collection and structure refinement details are summarized in Table 2 ▸. The hydrogen atoms attached to the nitro­gen atom (N5) of the amide linker and the carbon atoms (C30, C31, C32, C33) of the crown ether were placed in the locations derived from a difference map, while others were placed in calculated positions (C—H = 0.95, 0.99 Å for aryl and methine H atoms, respectively). Hydrogen atoms were refined using a riding model with fixed isotropic displacement parameters of *U*_iso_(H) = 1.2*U*_eq_(C). One outlier was omitted in the last cycles of refinement.

## Supplementary Material

Crystal structure: contains datablock(s) I. DOI: 10.1107/S2056989017007745/qm2115sup1.cif

Structure factors: contains datablock(s) I. DOI: 10.1107/S2056989017007745/qm2115Isup2.hkl

CCDC reference: 1552184

Additional supporting information:  crystallographic information; 3D view; checkCIF report

## Figures and Tables

**Table 1:** Hydrogen-bond geometry (Å, °)

*D*—H⋯*A*	*D*—H	H⋯*A*	*D*⋯*A*	*D*—H⋯*A*
N5—H5*B*⋯O2	0.93 (3)	1.99 (3)	2.866 (2)	156 (2)

**Table 2:** Experimental details

Crystal data	
Chemical formula	[Co(C_57_H_52_N_6_O_6_)]
*M*_r_	975.97
Crystal system, space group	Monoclinic, *P*2_1_/*n*
Temperature (K)	100
*a*, *b*, *c* (Å)	17.2445 (6), 14.1398 (5), 19.6452 (7)
β (°)	93.3307 (12)
*V* (Å^3^)	4782.1 (3)
*Z*	4
Radiation type	Mo *K*α
μ (mm^−1^)	0.42
Crystal size (mm)	0.37 × 0.20 × 0.06

Data collection	
Diffractometer	Bruker D8 QUEST System
Absorption correction	Multi-scan (*SADABS*; Bruker, 2013 ▸)
*T*_min_, *T*_max_	0.904, 0.975
No. of measured, independent and observed [*I* > 2σ(*I*)] reflections	70434, 10590, 8774
*R*_int_	0.062
(sin θ/λ)_max_ (Å^−1^)	0.643

Refinement	
*R*[*F* ^2^ > 2σ(*F* ^2^)], *wR*(*F* ^2^), *S*	0.043, 0.106, 1.06
No. of reflections	10590
No. of parameters	667
H-atom treatment	H atoms treated by a mixture of independent and constrained refinement
Δρ_max_, Δρ_min_ (e Å^−3^)	0.69, −0.43

Computer programs: *APEX2* (Bruker, 2013 ▸),*SAINT* and *XPREP* (Bruker, 2013 ▸), *SHELXT* (Sheldrick, 2015*a* ▸), *SHELXL2014* and *SHELXCIF2014* (Sheldrick, 2015*b* ▸), *XP* (Sheldrick, 2008 ▸) and *enCIFer* (Allen *et al.*, 2004 ▸).

## supplementary crystallographic information

### Crystal data

**Table:**

[Co(C_57_H_52_N_6_O_6_)]	*F*(000) = 2044
*M**_r_* = 975.97	*D*_x_ = 1.356 Mg m^−^^3^
Monoclinic, *P*2_1_/*n*	Mo *K*α radiation, λ = 0.71073 Å
*a* = 17.2445 (6) Å	Cell parameters from 9294 reflections
*b* = 14.1398 (5) Å	θ = 2.7–27.2°
*c* = 19.6452 (7) Å	µ = 0.42 mm^−^^1^
β = 93.3307 (12)°	*T* = 100 K
*V* = 4782.1 (3) Å^3^	Block, black
*Z* = 4	0.37 × 0.20 × 0.06 mm

### Data collection

**Table:**

Bruker D8 QUEST System diffractometer	8774 reflections with *I* > 2σ(*I*)
Radiation source: fine-focus sealed tube	*R*_int_ = 0.062
φ and ω scans	θ_max_ = 27.2°, θ_min_ = 2.7°
Absorption correction: multi-scan (SADABS; Sheldrick, 2008)	*h* = −22→22
*T*_min_ = 0.904, *T*_max_ = 0.975	*k* = −18→18
70434 measured reflections	*l* = −25→25
10590 independent reflections	

### Refinement

**Table:**

Refinement on *F*^2^	Primary atom site location: structure-invariant direct methods
Least-squares matrix: full	Secondary atom site location: difference Fourier map
*R*[*F*^2^ > 2σ(*F*^2^)] = 0.043	Hydrogen site location: mixed
*wR*(*F*^2^) = 0.106	H atoms treated by a mixture of independent and constrained refinement
*S* = 1.06	*w* = 1/[σ^2^(*F*_o_^2^) + (0.0377*P*)^2^ + 5.0698*P*] where *P* = (*F*_o_^2^ + 2*F*_c_^2^)/3
10590 reflections	(Δ/σ)_max_ = 0.001
667 parameters	Δρ_max_ = 0.69 e Å^−^^3^
0 restraints	Δρ_min_ = −0.43 e Å^−^^3^

### Special details

**Table:**

Geometry. All esds (except the esd in the dihedral angle between two l.s. planes) are estimated using the full covariance matrix. The cell esds are taken into account individually in the estimation of esds in distances, angles and torsion angles; correlations between esds in cell parameters are only used when they are defined by crystal symmetry. An approximate (isotropic) treatment of cell esds is used for estimating esds involving l.s. planes.
Refinement. Refinement of F^2^ against ALL reflections. The weighted R-factor wR and goodness of fit S are based on F^2^, conventional R-factors R are based on F, with F set to zero for negative F^2^. The threshold expression of F^2^ > 2sigma(F^2^) is used only for calculating R-factors(gt) etc. and is not relevant to the choice of reflections for refinement. R-factors based on F^2^ are statistically about twice as large as those based on F, and R- factors based on ALL data will be even larger.

### Fractional atomic coordinates and isotropic or equivalent isotropic displacement parameters (Å^2^)

**Table:**

	*x*	*y*	*z*	*U*_iso_*/*U*_eq_	
Co1	0.77071 (2)	0.20877 (2)	0.81653 (2)	0.01301 (7)	
N1	0.82538 (9)	0.21197 (11)	0.90768 (8)	0.0141 (3)	
N2	0.67472 (9)	0.16966 (11)	0.85777 (8)	0.0145 (3)	
N3	0.71366 (9)	0.21920 (11)	0.72734 (8)	0.0141 (3)	
N4	0.86690 (9)	0.24988 (11)	0.77621 (8)	0.0152 (3)	
C101	0.89945 (11)	0.24414 (14)	0.92504 (9)	0.0166 (4)	
C102	0.79657 (11)	0.18437 (14)	0.96887 (9)	0.0159 (4)	
C103	0.66772 (11)	0.13693 (13)	0.92345 (9)	0.0151 (4)	
C104	0.60177 (11)	0.15961 (13)	0.82619 (9)	0.0155 (4)	
C105	0.63444 (11)	0.21165 (13)	0.71265 (9)	0.0154 (4)	
C106	0.74468 (11)	0.23415 (13)	0.66505 (9)	0.0149 (4)	
C107	0.87938 (11)	0.25847 (14)	0.70782 (9)	0.0161 (4)	
C108	0.93724 (11)	0.27208 (14)	0.80922 (10)	0.0165 (4)	
C201	0.91561 (12)	0.23959 (16)	0.99755 (10)	0.0217 (4)	
H(BA	0.9622	0.2589	1.0218	0.026*	
C202	0.85233 (12)	0.20276 (15)	1.02461 (10)	0.0214 (4)	
H(BB	0.8457	0.1911	1.0716	0.026*	
C203	0.59031 (11)	0.10269 (14)	0.93144 (10)	0.0189 (4)	
H(BC	0.5713	0.0753	0.9714	0.023*	
C204	0.54965 (11)	0.11668 (14)	0.87155 (10)	0.0180 (4)	
H(BD	0.4967	0.1011	0.8613	0.022*	
C205	0.61636 (11)	0.22583 (14)	0.64096 (9)	0.0183 (4)	
H(BE	0.5660	0.2266	0.6186	0.022*	
C206	0.68428 (11)	0.23789 (14)	0.61139 (9)	0.0176 (4)	
H(BF	0.6910	0.2471	0.5642	0.021*	
C207	0.95868 (11)	0.28399 (15)	0.69821 (10)	0.0218 (4)	
H(BG	0.9818	0.2918	0.6558	0.026*	
C208	0.99406 (12)	0.29471 (16)	0.76082 (10)	0.0221 (4)	
H(BH	1.0463	0.3135	0.7710	0.027*	
C301	0.95317 (11)	0.27274 (14)	0.87929 (9)	0.0166 (4)	
C302	0.72418 (11)	0.14436 (13)	0.97660 (9)	0.0154 (4)	
C303	0.58063 (11)	0.18478 (13)	0.75899 (9)	0.0156 (4)	
C304	0.82307 (11)	0.24882 (13)	0.65463 (9)	0.0158 (4)	
C1	1.03134 (11)	0.30727 (15)	0.90615 (9)	0.0190 (4)	
C2	1.03696 (12)	0.39518 (16)	0.93759 (10)	0.0241 (4)	
H2A	0.9909	0.4297	0.9449	0.029*	
C3	1.10865 (13)	0.43372 (16)	0.95862 (11)	0.0265 (5)	
H3A	1.1116	0.4936	0.9806	0.032*	
C4	1.17555 (12)	0.38379 (16)	0.94717 (11)	0.0255 (5)	
H4A	1.2248	0.4107	0.9597	0.031*	
C5	1.17140 (12)	0.29514 (16)	0.91776 (10)	0.0228 (4)	
H5A	1.2177	0.2609	0.9112	0.027*	
C6	1.09946 (11)	0.25552 (15)	0.89764 (10)	0.0193 (4)	
C7	0.70432 (11)	0.11388 (15)	1.04666 (9)	0.0182 (4)	
C8	0.67899 (13)	0.18032 (17)	1.09286 (11)	0.0291 (5)	
H8A	0.6739	0.2448	1.0797	0.035*	
C9	0.66109 (14)	0.15283 (19)	1.15802 (11)	0.0335 (5)	
H9A	0.6451	0.1988	1.1896	0.040*	
C10	0.66645 (13)	0.05914 (19)	1.17709 (11)	0.0313 (5)	
H10A	0.6531	0.0405	1.2213	0.038*	
C11	0.69126 (13)	−0.00749 (18)	1.13190 (11)	0.0299 (5)	
H11A	0.6950	−0.0721	1.1450	0.036*	
C12	0.71088 (12)	0.02048 (16)	1.06662 (10)	0.0236 (4)	
H12A	0.7289	−0.0253	1.0358	0.028*	
C13	0.49628 (11)	0.17977 (14)	0.73654 (9)	0.0173 (4)	
C14	0.44431 (12)	0.24220 (15)	0.76426 (10)	0.0213 (4)	
H14A	0.4630	0.2885	0.7962	0.026*	
C15	0.36532 (12)	0.23753 (16)	0.74581 (11)	0.0253 (5)	
H15A	0.3302	0.2794	0.7660	0.030*	
C16	0.33789 (13)	0.17188 (18)	0.69811 (11)	0.0290 (5)	
H16A	0.2842	0.1699	0.6843	0.035*	
C17	0.38857 (13)	0.10962 (19)	0.67089 (12)	0.0337 (5)	
H17A	0.3696	0.0639	0.6386	0.040*	
C18	0.46763 (12)	0.11270 (17)	0.69006 (11)	0.0271 (5)	
H18A	0.5021	0.0687	0.6712	0.033*	
C19	0.84853 (11)	0.25600 (14)	0.58351 (9)	0.0162 (4)	
C20	0.87286 (12)	0.34129 (15)	0.55670 (10)	0.0233 (4)	
H20A	0.8704	0.3977	0.5828	0.028*	
C21	0.90072 (13)	0.34417 (17)	0.49178 (11)	0.0277 (5)	
H21A	0.9180	0.4024	0.4739	0.033*	
C22	0.90336 (13)	0.26276 (17)	0.45314 (11)	0.0281 (5)	
H22A	0.9221	0.2652	0.4086	0.034*	
C23	0.87903 (12)	0.17848 (17)	0.47881 (11)	0.0273 (5)	
H23A	0.8808	0.1226	0.4521	0.033*	
C24	0.85176 (12)	0.17490 (15)	0.54403 (10)	0.0212 (4)	
H24A	0.8351	0.1162	0.5617	0.025*	
N5	1.09580 (10)	0.16211 (13)	0.87059 (9)	0.0219 (4)	
H5B	1.0535 (17)	0.124 (2)	0.8796 (14)	0.043 (8)*	
C25	1.13590 (11)	0.13717 (15)	0.81420 (10)	0.0209 (4)	
O1	1.17389 (8)	0.19470 (11)	0.78285 (8)	0.0257 (3)	
N6	1.12918 (10)	0.04417 (13)	0.79513 (9)	0.0226 (4)	
O2	0.98449 (8)	0.01154 (11)	0.87015 (8)	0.0262 (3)	
O3	0.79383 (8)	0.05063 (10)	0.78948 (8)	0.0253 (3)	
O4	0.76136 (9)	−0.07052 (11)	0.66442 (8)	0.0298 (4)	
O5	0.87241 (9)	−0.04949 (11)	0.55450 (8)	0.0309 (4)	
O6	1.04706 (9)	−0.02582 (11)	0.66271 (8)	0.0288 (3)	
C26	1.11134 (12)	−0.03147 (15)	0.84266 (11)	0.0248 (4)	
H26A	1.1258	−0.0100	0.8897	0.030*	
H26B	1.1438	−0.0872	0.8333	0.030*	
C27	1.02716 (12)	−0.06149 (15)	0.83898 (12)	0.0261 (5)	
H27A	1.0080	−0.0701	0.7909	0.031*	
H27B	1.0212	−0.1220	0.8634	0.031*	
C28	0.90267 (12)	−0.00076 (16)	0.86277 (11)	0.0251 (5)	
H28A	0.8774	0.0433	0.8937	0.030*	
H28B	0.8894	−0.0661	0.8760	0.030*	
C29	0.87176 (12)	0.01736 (16)	0.78963 (11)	0.0265 (5)	
H29A	0.9046	0.0650	0.7682	0.032*	
H29B	0.8736	−0.0419	0.7629	0.032*	
C30	0.73435 (13)	−0.02000 (16)	0.77704 (13)	0.0302 (5)	
H30A	0.7553 (14)	−0.0834 (19)	0.7934 (12)	0.031 (7)*	
H30B	0.6907 (16)	−0.0014 (19)	0.8086 (14)	0.040 (7)*	
C31	0.70500 (14)	−0.02511 (17)	0.70410 (14)	0.0331 (5)	
H31A	0.6524 (17)	−0.067 (2)	0.7009 (14)	0.049 (8)*	
H31B	0.6940 (17)	0.043 (2)	0.6862 (14)	0.046 (8)*	
C32	0.74101 (14)	−0.06236 (17)	0.59341 (13)	0.0305 (5)	
H32A	0.6896 (15)	−0.0967 (19)	0.5824 (13)	0.034 (7)*	
H32B	0.7317 (14)	0.0051 (19)	0.5820 (13)	0.031 (7)*	
C33	0.80347 (15)	−0.10470 (17)	0.55274 (12)	0.0315 (5)	
H33A	0.8161 (14)	−0.1692 (19)	0.5687 (12)	0.030 (6)*	
H33B	0.7829 (16)	−0.105 (2)	0.5014 (14)	0.044 (8)*	
C34	0.93009 (14)	−0.07851 (16)	0.60548 (12)	0.0294 (5)	
H34A	0.9554	−0.1377	0.5915	0.035*	
H34B	0.9062	−0.0896	0.6494	0.035*	
C35	0.98875 (14)	0.00081 (17)	0.61245 (12)	0.0316 (5)	
H35A	1.0121	0.0120	0.5683	0.038*	
H35B	0.9631	0.0599	0.6262	0.038*	
C36	1.10801 (13)	0.04208 (16)	0.66854 (11)	0.0287 (5)	
H36A	1.0860	0.1062	0.6736	0.034*	
H36B	1.1377	0.0412	0.6269	0.034*	
C37	1.16125 (13)	0.01862 (16)	0.73010 (11)	0.0262 (5)	
H37A	1.1722	−0.0501	0.7301	0.031*	
H37B	1.2112	0.0522	0.7262	0.031*	

### Atomic displacement parameters (Å^2^)

**Table:**

	*U*^11^	*U*^22^	*U*^33^	*U*^12^	*U*^13^	*U*^23^
Co1	0.01207 (12)	0.01794 (13)	0.00923 (12)	−0.00057 (10)	0.00228 (9)	0.00073 (10)
N1	0.0128 (7)	0.0173 (8)	0.0125 (7)	0.0002 (6)	0.0035 (6)	0.0008 (6)
N2	0.0147 (8)	0.0176 (8)	0.0116 (7)	0.0007 (6)	0.0030 (6)	0.0007 (6)
N3	0.0147 (7)	0.0165 (8)	0.0114 (7)	−0.0013 (6)	0.0034 (6)	−0.0003 (6)
N4	0.0156 (8)	0.0185 (8)	0.0115 (7)	−0.0008 (6)	0.0018 (6)	0.0011 (6)
C101	0.0145 (9)	0.0210 (10)	0.0142 (9)	0.0012 (7)	0.0006 (7)	0.0012 (7)
C102	0.0158 (9)	0.0190 (9)	0.0133 (8)	0.0031 (7)	0.0026 (7)	0.0012 (7)
C103	0.0156 (9)	0.0174 (9)	0.0129 (8)	0.0006 (7)	0.0049 (7)	0.0008 (7)
C104	0.0135 (9)	0.0184 (9)	0.0149 (9)	−0.0005 (7)	0.0030 (7)	−0.0014 (7)
C105	0.0158 (9)	0.0170 (9)	0.0132 (8)	−0.0003 (7)	0.0010 (7)	−0.0012 (7)
C106	0.0194 (9)	0.0149 (9)	0.0104 (8)	−0.0010 (7)	0.0021 (7)	0.0001 (7)
C107	0.0156 (9)	0.0191 (9)	0.0139 (9)	−0.0012 (7)	0.0044 (7)	0.0011 (7)
C108	0.0136 (9)	0.0206 (10)	0.0156 (9)	−0.0018 (7)	0.0027 (7)	0.0019 (7)
C201	0.0181 (10)	0.0327 (12)	0.0138 (9)	−0.0012 (8)	−0.0026 (7)	0.0020 (8)
C202	0.0200 (10)	0.0322 (11)	0.0120 (9)	0.0005 (8)	0.0002 (7)	0.0033 (8)
C203	0.0174 (9)	0.0234 (10)	0.0162 (9)	−0.0008 (8)	0.0051 (7)	0.0042 (8)
C204	0.0149 (9)	0.0223 (10)	0.0172 (9)	−0.0011 (8)	0.0037 (7)	0.0022 (8)
C205	0.0184 (9)	0.0226 (10)	0.0137 (9)	−0.0001 (8)	−0.0002 (7)	−0.0003 (7)
C206	0.0214 (10)	0.0208 (10)	0.0107 (8)	−0.0016 (8)	0.0012 (7)	−0.0002 (7)
C207	0.0187 (10)	0.0310 (11)	0.0161 (9)	−0.0029 (8)	0.0047 (7)	0.0039 (8)
C208	0.0151 (9)	0.0332 (12)	0.0184 (9)	−0.0045 (8)	0.0036 (7)	0.0053 (9)
C301	0.0138 (9)	0.0205 (10)	0.0156 (9)	−0.0005 (7)	0.0003 (7)	0.0008 (7)
C302	0.0169 (9)	0.0173 (9)	0.0123 (8)	0.0022 (7)	0.0033 (7)	0.0012 (7)
C303	0.0140 (9)	0.0175 (9)	0.0155 (9)	0.0002 (7)	0.0015 (7)	−0.0012 (7)
C304	0.0197 (9)	0.0164 (9)	0.0115 (8)	−0.0012 (7)	0.0044 (7)	0.0007 (7)
C1	0.0169 (9)	0.0278 (11)	0.0124 (9)	−0.0030 (8)	0.0007 (7)	0.0041 (8)
C2	0.0183 (10)	0.0320 (12)	0.0218 (10)	0.0000 (9)	0.0009 (8)	0.0007 (9)
C3	0.0264 (11)	0.0292 (12)	0.0237 (10)	−0.0060 (9)	−0.0008 (9)	−0.0020 (9)
C4	0.0197 (10)	0.0343 (12)	0.0220 (10)	−0.0077 (9)	−0.0028 (8)	0.0054 (9)
C5	0.0156 (9)	0.0323 (12)	0.0203 (10)	−0.0024 (8)	−0.0010 (8)	0.0050 (9)
C6	0.0176 (9)	0.0257 (10)	0.0147 (9)	−0.0024 (8)	0.0010 (7)	0.0048 (8)
C7	0.0118 (9)	0.0304 (11)	0.0125 (9)	−0.0024 (8)	0.0011 (7)	0.0023 (8)
C8	0.0333 (12)	0.0331 (12)	0.0220 (11)	−0.0002 (10)	0.0106 (9)	−0.0002 (9)
C9	0.0306 (12)	0.0528 (16)	0.0184 (10)	−0.0029 (11)	0.0118 (9)	−0.0069 (10)
C10	0.0229 (11)	0.0560 (16)	0.0153 (10)	−0.0056 (10)	0.0026 (8)	0.0089 (10)
C11	0.0258 (11)	0.0387 (13)	0.0248 (11)	−0.0037 (10)	−0.0012 (9)	0.0135 (10)
C12	0.0229 (10)	0.0292 (11)	0.0189 (10)	−0.0016 (9)	0.0023 (8)	0.0026 (8)
C13	0.0150 (9)	0.0239 (10)	0.0132 (9)	−0.0031 (7)	0.0020 (7)	0.0022 (7)
C14	0.0221 (10)	0.0226 (10)	0.0194 (10)	0.0002 (8)	0.0040 (8)	0.0024 (8)
C15	0.0203 (10)	0.0272 (11)	0.0291 (11)	0.0034 (8)	0.0072 (8)	0.0082 (9)
C16	0.0175 (10)	0.0417 (13)	0.0277 (11)	−0.0043 (9)	−0.0008 (8)	0.0105 (10)
C17	0.0258 (12)	0.0440 (14)	0.0309 (12)	−0.0098 (10)	−0.0028 (9)	−0.0102 (11)
C18	0.0212 (10)	0.0326 (12)	0.0277 (11)	−0.0008 (9)	0.0026 (8)	−0.0092 (9)
C19	0.0137 (9)	0.0223 (10)	0.0126 (9)	−0.0004 (7)	0.0021 (7)	0.0018 (7)
C20	0.0272 (11)	0.0238 (11)	0.0197 (10)	0.0022 (9)	0.0074 (8)	0.0020 (8)
C21	0.0299 (12)	0.0307 (12)	0.0232 (11)	0.0008 (9)	0.0083 (9)	0.0099 (9)
C22	0.0259 (11)	0.0437 (14)	0.0155 (10)	−0.0003 (10)	0.0081 (8)	−0.0020 (9)
C23	0.0238 (11)	0.0371 (13)	0.0215 (10)	−0.0016 (9)	0.0058 (8)	−0.0104 (9)
C24	0.0190 (10)	0.0245 (10)	0.0202 (10)	−0.0012 (8)	0.0029 (8)	−0.0012 (8)
N5	0.0174 (8)	0.0244 (9)	0.0243 (9)	−0.0029 (7)	0.0032 (7)	0.0026 (7)
C25	0.0150 (9)	0.0265 (11)	0.0210 (10)	0.0012 (8)	−0.0010 (8)	0.0034 (8)
O1	0.0225 (7)	0.0285 (8)	0.0264 (8)	−0.0028 (6)	0.0044 (6)	0.0041 (6)
N6	0.0197 (9)	0.0234 (9)	0.0250 (9)	0.0014 (7)	0.0033 (7)	0.0032 (7)
O2	0.0188 (7)	0.0254 (8)	0.0347 (8)	−0.0006 (6)	0.0040 (6)	−0.0012 (7)
O3	0.0188 (7)	0.0204 (7)	0.0371 (9)	0.0019 (6)	0.0033 (6)	−0.0022 (6)
O4	0.0266 (8)	0.0276 (8)	0.0346 (9)	0.0043 (7)	−0.0046 (7)	−0.0007 (7)
O5	0.0346 (9)	0.0254 (8)	0.0317 (8)	−0.0043 (7)	−0.0073 (7)	0.0071 (7)
O6	0.0299 (8)	0.0273 (8)	0.0284 (8)	−0.0018 (7)	−0.0050 (7)	0.0039 (7)
C26	0.0216 (10)	0.0230 (11)	0.0297 (11)	0.0023 (8)	0.0012 (8)	0.0047 (9)
C27	0.0231 (11)	0.0208 (10)	0.0346 (12)	0.0016 (8)	0.0036 (9)	0.0025 (9)
C28	0.0200 (10)	0.0237 (11)	0.0320 (12)	−0.0002 (8)	0.0058 (9)	0.0027 (9)
C29	0.0221 (10)	0.0264 (11)	0.0316 (12)	0.0065 (9)	0.0058 (9)	0.0029 (9)
C30	0.0249 (11)	0.0192 (11)	0.0472 (14)	−0.0015 (9)	0.0089 (10)	−0.0010 (10)
C31	0.0228 (11)	0.0211 (11)	0.0551 (16)	−0.0006 (9)	−0.0008 (10)	−0.0061 (11)
C32	0.0300 (12)	0.0213 (11)	0.0386 (13)	−0.0044 (9)	−0.0127 (10)	0.0060 (10)
C33	0.0398 (13)	0.0223 (12)	0.0311 (12)	−0.0064 (10)	−0.0083 (10)	0.0032 (9)
C34	0.0335 (12)	0.0252 (11)	0.0289 (12)	0.0022 (9)	−0.0028 (9)	0.0038 (9)
C35	0.0369 (13)	0.0270 (12)	0.0297 (12)	0.0003 (10)	−0.0074 (10)	0.0049 (9)
C36	0.0318 (12)	0.0264 (11)	0.0278 (11)	−0.0038 (9)	0.0024 (9)	0.0026 (9)
C37	0.0236 (11)	0.0283 (11)	0.0269 (11)	0.0020 (9)	0.0038 (9)	−0.0001 (9)

### Geometric parameters (Å, º)

**Table:**

Co1—N2	1.9641 (15)	C13—C14	1.391 (3)
Co1—N3	1.9645 (15)	C14—C15	1.391 (3)
Co1—N4	1.9671 (16)	C14—H14A	0.9500
Co1—N1	1.9751 (15)	C15—C16	1.383 (3)
Co1—O3	2.3380 (15)	C15—H15A	0.9500
N1—C101	1.380 (2)	C16—C17	1.371 (3)
N1—C102	1.383 (2)	C16—H16A	0.9500
N2—C104	1.377 (2)	C17—C18	1.394 (3)
N2—C103	1.383 (2)	C17—H17A	0.9500
N3—C106	1.380 (2)	C18—H18A	0.9500
N3—C105	1.384 (2)	C19—C24	1.387 (3)
N4—C108	1.378 (2)	C19—C20	1.391 (3)
N4—C107	1.378 (2)	C20—C21	1.389 (3)
C101—C301	1.388 (3)	C20—H20A	0.9500
C101—C201	1.437 (3)	C21—C22	1.381 (3)
C102—C302	1.387 (3)	C21—H21A	0.9500
C102—C202	1.438 (3)	C22—C23	1.369 (3)
C103—C302	1.390 (3)	C22—H22A	0.9500
C103—C203	1.437 (3)	C23—C24	1.391 (3)
C104—C303	1.395 (3)	C23—H23A	0.9500
C104—C204	1.437 (3)	C24—H24A	0.9500
C105—C303	1.390 (3)	N5—C25	1.385 (3)
C105—C205	1.439 (3)	N5—H5B	0.93 (3)
C106—C304	1.394 (3)	C25—O1	1.232 (2)
C106—C206	1.439 (3)	C25—N6	1.370 (3)
C107—C304	1.391 (3)	N6—C26	1.464 (3)
C107—C207	1.437 (3)	N6—C37	1.466 (3)
C108—C301	1.388 (3)	O2—C28	1.421 (2)
C108—C208	1.440 (3)	O2—C27	1.427 (3)
C201—C202	1.346 (3)	O3—C29	1.424 (2)
C201—H(BA	0.9500	O3—C30	1.442 (3)
C202—H(BB	0.9500	O4—C32	1.423 (3)
C203—C204	1.349 (3)	O4—C31	1.433 (3)
C203—H(BC	0.9500	O5—C33	1.421 (3)
C204—H(BD	0.9500	O5—C34	1.430 (3)
C205—C206	1.348 (3)	O6—C35	1.419 (3)
C205—H(BE	0.9500	O6—C36	1.423 (3)
C206—H(BF	0.9500	C26—C27	1.510 (3)
C207—C208	1.349 (3)	C26—H26A	0.9900
C207—H(BG	0.9500	C26—H26B	0.9900
C208—H(BH	0.9500	C27—H27A	0.9900
C301—C1	1.500 (3)	C27—H27B	0.9900
C302—C7	1.501 (2)	C28—C29	1.525 (3)
C303—C13	1.497 (3)	C28—H28A	0.9900
C304—C19	1.492 (2)	C28—H28B	0.9900
C1—C2	1.389 (3)	C29—H29A	0.9900
C1—C6	1.402 (3)	C29—H29B	0.9900
C2—C3	1.392 (3)	C30—C31	1.494 (4)
C2—H2A	0.9500	C30—H30A	1.01 (3)
C3—C4	1.382 (3)	C30—H30B	1.04 (3)
C3—H3A	0.9500	C31—H31A	1.08 (3)
C4—C5	1.380 (3)	C31—H31B	1.04 (3)
C4—H4A	0.9500	C32—C33	1.502 (4)
C5—C6	1.397 (3)	C32—H32A	1.02 (3)
C5—H5A	0.9500	C32—H32B	0.99 (3)
C6—N5	1.424 (3)	C33—H33A	0.98 (3)
C7—C12	1.380 (3)	C33—H33B	1.05 (3)
C7—C8	1.394 (3)	C34—C35	1.511 (3)
C8—C9	1.390 (3)	C34—H34A	0.9900
C8—H8A	0.9500	C34—H34B	0.9900
C9—C10	1.378 (4)	C35—H35A	0.9900
C9—H9A	0.9500	C35—H35B	0.9900
C10—C11	1.379 (4)	C36—C37	1.512 (3)
C10—H10A	0.9500	C36—H36A	0.9900
C11—C12	1.402 (3)	C36—H36B	0.9900
C11—H11A	0.9500	C37—H37A	0.9900
C12—H12A	0.9500	C37—H37B	0.9900
C13—C18	1.387 (3)		
			
N2—Co1—N3	89.92 (6)	C15—C14—C13	120.7 (2)
N2—Co1—N4	179.03 (7)	C15—C14—H14A	119.6
N3—Co1—N4	90.51 (6)	C13—C14—H14A	119.6
N2—Co1—N1	90.11 (6)	C16—C15—C14	120.0 (2)
N3—Co1—N1	174.16 (7)	C16—C15—H15A	120.0
N4—Co1—N1	89.37 (6)	C14—C15—H15A	120.0
N2—Co1—O3	89.05 (6)	C17—C16—C15	119.7 (2)
N3—Co1—O3	87.26 (6)	C17—C16—H16A	120.2
N4—Co1—O3	91.84 (6)	C15—C16—H16A	120.2
N1—Co1—O3	98.58 (6)	C16—C17—C18	120.7 (2)
C101—N1—C102	104.61 (15)	C16—C17—H17A	119.7
C101—N1—Co1	128.17 (12)	C18—C17—H17A	119.7
C102—N1—Co1	127.21 (13)	C13—C18—C17	120.3 (2)
C104—N2—C103	105.00 (15)	C13—C18—H18A	119.9
C104—N2—Co1	127.80 (12)	C17—C18—H18A	119.9
C103—N2—Co1	126.91 (12)	C24—C19—C20	118.83 (18)
C106—N3—C105	104.82 (15)	C24—C19—C304	119.40 (17)
C106—N3—Co1	127.06 (13)	C20—C19—C304	121.69 (18)
C105—N3—Co1	128.11 (12)	C21—C20—C19	120.1 (2)
C108—N4—C107	104.81 (15)	C21—C20—H20A	120.0
C108—N4—Co1	128.14 (12)	C19—C20—H20A	120.0
C107—N4—Co1	126.99 (13)	C22—C21—C20	120.3 (2)
N1—C101—C301	125.38 (17)	C22—C21—H21A	119.9
N1—C101—C201	110.72 (16)	C20—C21—H21A	119.9
C301—C101—C201	123.83 (18)	C23—C22—C21	120.14 (19)
N1—C102—C302	125.54 (17)	C23—C22—H22A	119.9
N1—C102—C202	110.65 (16)	C21—C22—H22A	119.9
C302—C102—C202	123.81 (17)	C22—C23—C24	119.9 (2)
N2—C103—C302	125.49 (17)	C22—C23—H23A	120.1
N2—C103—C203	110.29 (16)	C24—C23—H23A	120.1
C302—C103—C203	123.92 (17)	C19—C24—C23	120.8 (2)
N2—C104—C303	125.56 (17)	C19—C24—H24A	119.6
N2—C104—C204	110.62 (16)	C23—C24—H24A	119.6
C303—C104—C204	123.79 (17)	C25—N5—C6	121.42 (17)
N3—C105—C303	124.96 (17)	C25—N5—H5B	116.2 (17)
N3—C105—C205	110.39 (16)	C6—N5—H5B	118.6 (17)
C303—C105—C205	124.36 (17)	O1—C25—N6	122.40 (19)
N3—C106—C304	125.58 (17)	O1—C25—N5	122.4 (2)
N3—C106—C206	110.73 (16)	N6—C25—N5	115.14 (18)
C304—C106—C206	123.61 (17)	C25—N6—C26	122.96 (17)
N4—C107—C304	125.58 (17)	C25—N6—C37	116.39 (17)
N4—C107—C207	110.71 (16)	C26—N6—C37	118.65 (17)
C304—C107—C207	123.64 (17)	C28—O2—C27	113.68 (16)
N4—C108—C301	125.89 (17)	C29—O3—C30	115.69 (17)
N4—C108—C208	110.73 (16)	C29—O3—Co1	119.28 (13)
C301—C108—C208	123.38 (18)	C30—O3—Co1	124.87 (12)
C202—C201—C101	107.08 (17)	C32—O4—C31	111.13 (18)
C202—C201—H(BA	126.5	C33—O5—C34	113.97 (17)
C101—C201—H(BA	126.5	C35—O6—C36	111.54 (17)
C201—C202—C102	106.90 (17)	N6—C26—C27	114.33 (18)
C201—C202—H(BB	126.6	N6—C26—H26A	108.7
C102—C202—H(BB	126.6	C27—C26—H26A	108.7
C204—C203—C103	107.12 (17)	N6—C26—H26B	108.7
C204—C203—H(BC	126.4	C27—C26—H26B	108.7
C103—C203—H(BC	126.4	H26A—C26—H26B	107.6
C203—C204—C104	106.91 (17)	O2—C27—C26	107.12 (18)
C203—C204—H(BD	126.5	O2—C27—H27A	110.3
C104—C204—H(BD	126.5	C26—C27—H27A	110.3
C206—C205—C105	107.17 (17)	O2—C27—H27B	110.3
C206—C205—H(BE	126.4	C26—C27—H27B	110.3
C105—C205—H(BE	126.4	H27A—C27—H27B	108.5
C205—C206—C106	106.83 (16)	O2—C28—C29	111.49 (17)
C205—C206—H(BF	126.6	O2—C28—H28A	109.3
C106—C206—H(BF	126.6	C29—C28—H28A	109.3
C208—C207—C107	106.97 (17)	O2—C28—H28B	109.3
C208—C207—H(BG	126.5	C29—C28—H28B	109.3
C107—C207—H(BG	126.5	H28A—C28—H28B	108.0
C207—C208—C108	106.73 (17)	O3—C29—C28	109.62 (17)
C207—C208—H(BH	126.6	O3—C29—H29A	109.7
C108—C208—H(BH	126.6	C28—C29—H29A	109.7
C101—C301—C108	122.64 (18)	O3—C29—H29B	109.7
C101—C301—C1	119.14 (17)	C28—C29—H29B	109.7
C108—C301—C1	118.22 (16)	H29A—C29—H29B	108.2
C102—C302—C103	122.98 (17)	O3—C30—C31	113.2 (2)
C102—C302—C7	118.19 (17)	O3—C30—H30A	108.8 (14)
C103—C302—C7	118.70 (16)	C31—C30—H30A	110.9 (14)
C105—C303—C104	122.80 (17)	O3—C30—H30B	104.9 (15)
C105—C303—C13	119.61 (16)	C31—C30—H30B	111.6 (15)
C104—C303—C13	117.57 (16)	H30A—C30—H30B	107 (2)
C107—C304—C106	123.00 (17)	O4—C31—C30	109.93 (19)
C107—C304—C19	117.78 (16)	O4—C31—H31A	108.5 (16)
C106—C304—C19	119.21 (17)	C30—C31—H31A	108.6 (15)
C2—C1—C6	118.86 (18)	O4—C31—H31B	110.4 (16)
C2—C1—C301	119.05 (18)	C30—C31—H31B	108.9 (16)
C6—C1—C301	122.02 (18)	H31A—C31—H31B	110 (2)
C1—C2—C3	121.3 (2)	O4—C32—C33	110.20 (19)
C1—C2—H2A	119.3	O4—C32—H32A	109.3 (14)
C3—C2—H2A	119.3	C33—C32—H32A	109.7 (14)
C4—C3—C2	119.2 (2)	O4—C32—H32B	109.1 (15)
C4—C3—H3A	120.4	C33—C32—H32B	112.1 (15)
C2—C3—H3A	120.4	H32A—C32—H32B	106 (2)
C5—C4—C3	120.6 (2)	O5—C33—C32	113.13 (19)
C5—C4—H4A	119.7	O5—C33—H33A	109.3 (15)
C3—C4—H4A	119.7	C32—C33—H33A	110.6 (14)
C4—C5—C6	120.4 (2)	O5—C33—H33B	105.1 (15)
C4—C5—H5A	119.8	C32—C33—H33B	107.4 (15)
C6—C5—H5A	119.8	H33A—C33—H33B	111 (2)
C5—C6—C1	119.56 (19)	O5—C34—C35	106.57 (18)
C5—C6—N5	119.80 (18)	O5—C34—H34A	110.4
C1—C6—N5	120.61 (18)	C35—C34—H34A	110.4
C12—C7—C8	118.91 (18)	O5—C34—H34B	110.4
C12—C7—C302	121.05 (18)	C35—C34—H34B	110.4
C8—C7—C302	120.03 (19)	H34A—C34—H34B	108.6
C9—C8—C7	120.4 (2)	O6—C35—C34	108.04 (18)
C9—C8—H8A	119.8	O6—C35—H35A	110.1
C7—C8—H8A	119.8	C34—C35—H35A	110.1
C10—C9—C8	120.3 (2)	O6—C35—H35B	110.1
C10—C9—H9A	119.9	C34—C35—H35B	110.1
C8—C9—H9A	119.9	H35A—C35—H35B	108.4
C9—C10—C11	120.0 (2)	O6—C36—C37	109.12 (18)
C9—C10—H10A	120.0	O6—C36—H36A	109.9
C11—C10—H10A	120.0	C37—C36—H36A	109.9
C10—C11—C12	119.8 (2)	O6—C36—H36B	109.9
C10—C11—H11A	120.1	C37—C36—H36B	109.9
C12—C11—H11A	120.1	H36A—C36—H36B	108.3
C7—C12—C11	120.6 (2)	N6—C37—C36	113.61 (18)
C7—C12—H12A	119.7	N6—C37—H37A	108.8
C11—C12—H12A	119.7	C36—C37—H37A	108.8
C18—C13—C14	118.59 (19)	N6—C37—H37B	108.8
C18—C13—C303	121.95 (18)	C36—C37—H37B	108.8
C14—C13—C303	119.44 (18)	H37A—C37—H37B	107.7

### Hydrogen-bond geometry (Å, º)

**Table:**

*D*—H···*A*	*D*—H	H···*A*	*D*···*A*	*D*—H···*A*
N5—H5*B*···O2	0.93 (3)	1.99 (3)	2.866 (2)	156 (2)
